# Introducing the SLI debate

**DOI:** 10.1111/1460-6984.12119

**Published:** 2014-08-20

**Authors:** Susan Ebbels

It is my great pleasure to introduce this special issue on specific language impairment (SLI). The special issue re-examines the diagnostic criteria for SLI and questions whether the term ‘SLI’ should continue be used as a diagnostic label for children with ‘unexplained language problems’ (the term used by Bishop 2014 in her lead article).

This special issue has come about because of increasing dissatisfaction in many quarters with the wide variability in the diagnostic criteria used and the labels given to children with unexplained language problems. This variability is contributing to a lack of equity of access to services and limited recognition and understanding of children's language problems both by the general public and the scientific community (Bishop 2010). Recent population studies (e.g., Tomblin *et al*. 1996, Reilly *et al*. 2010) allow examination of the validity of the diagnostic criteria from a new perspective and thus reconsideration of our use of these criteria is timely. The debate about labels has been highlighted by the exclusion of SLI in the recently published *Diagnostic and Statistical Manual of Mental Disorders (DSM-5)* (American Psychiatric Association 2013), as recommended by the American Speech and Hearing Association (ASHA). ‘Language Disorder’ is used instead. The International Classification of Diseases and Related Health Problems (ICD-11), currently in preparation, is also unlikely to use ‘SLI’.

In the UK, a live debate entitled ‘What *is* Specific Language Impairment?’ in May 2012 raised several issues about diagnostic criteria, labels and services. The main issues regarding diagnosis were: the role of non-verbal IQ in diagnosis of language problems, differential diagnosis from autistic spectrum disorder (ASD) and the label that should be used for children with unexplained language difficulties. The debate also revealed that diagnostic labels and criteria were being used creatively in disputes over access to services both by those seeking to obtain services for children (often parents and their lawyers) who could be accused of ‘diagnostic shopping’ and also by those seeking to deny services (often due to financial constraints) who may use particularly restrictive criteria in order to reduce the number of children qualifying for services (see also Wright 2014). Videos and slides from the debate can been viewed at http://www.moorhouseschool.co.uk/sli-debate/.

Following this live debate, discussions with the Royal College of Speech and Language Therapists (RCSLT) about how to broaden the debate led to development of the SLI webpage (http://www.rcslt.org/members/clinical_areas/SLI) and a series of articles in the *Bulletin* from April to October 2013 (Bishop 2013, Clark *et al*. 2013, Dockrell 2013, Ebbels 2013, Lascelles 2013, McCartney 2013, Norbury 2013, Slonims 2013) focusing on diagnosis of SLI, labels used, how to meet language needs in the classrooms and the parental perspective.

Widening the debate further was still high on the agenda, thus I was delighted to learn that Sheena Reilly had been invited to present the *IJLCD* winter lecture in December 2013 and she was proposing to talk about the diagnostic criteria and label of SLI. Meanwhile, the RALLI (Raising Awareness of Language Learning Impairments) team, who aim to raise public awareness of children with unexplained language problems (see http://www.youtube.com/RALLIcampaign), found that terminology was a major stumbling block. As a result of lack of progress in this area, Dorothy Bishop suggested to the *IJLCD* editorial board that a special issue on SLI with lead articles and commentaries from a range of perspectives would be a good way to move the debate forward. Thus, the special issue was born, with two lead articles: one from Bishop entitled ‘Ten questions about terminology for children with unexplained language problems’ and one from Reilly and colleagues based on her winter lecture entitled: ‘Specific language impairment: a convenient label for whom?’ Each article is followed by 10 commentaries from a range of experts from several different countries commenting from their various perspectives as academics, speech and language therapists, educational psychologists, special educational needs lawyers, and representatives of charities working for and with children with unexplained language problems and their parents. The discussion is then continued in a response article jointly authored by Reilly, Bishop and Bruce Tomblin entitled: ‘Terminological debate over language impairment in children: forward movement and sticking points’.

The articles and commentaries raise many important issues about the diagnosis of children with unexplained language problems. They discuss identification of a language problem and the validity of exclusionary criteria. They argue that the precise criteria used may need to be different for research versus identification of those who need access to services (Bellair *et al*. 2014, Reilly *et al*. 2014b). Indeed, Rutter (2014) discusses how diagnosis of a disorder is not tantamount to a need for treatment, and Lauchlan and Boyle (2014) state that there is little evidence that a diagnostic label dictates the intervention a child should receive.

In terms of diagnostic criteria, even the identification of a language problem is not straightforward. Should language tests be used and if so, which tests, with which cut-points? Reilly *et al*. (2014a) suggest using a cut-point of –1.25 SD, with children scoring < –1 SD also being monitored. Several commentators point out problems with this approach, e.g. many false-positives may be identified (Norbury 2014) and standardized tests are particularly unreliable for identifying bilingual children (Bellair *et al*. 2014). There were also concerns that limited resources would be severely stretched by the identification of so many children (Norbury 2014, Parsons *et al*. 2014). The addition of functional assessments is advocated by both lead articles and many commentators, not only to reduce false-positives, but also to ensure that children with functional difficulties have access to services (Grist and Hartshorne 2014, Norbury 2014, Snowling 2014, Whitehouse 2014). In terms of provision of services, some commentators stress the need to consider the holistic profile of the child, the range of needs and how these impact on each other (Bellair *et al*. 2014, Strudwick and Bauer 2014).

The validity and usefulness of exclusionary criteria is a major focus of the articles in this special issue. While both lead articles recognize there may be a place for exclusionary criteria in some research studies (although they stress that these ‘pure cases’ will not then be representative of children in clinical contexts), they both argue that use of most exclusionary criteria is probably not justified for deciding who should receive intervention. Most commentators agree. Indeed, Dockrell and Lindsay (2014), see dropping exclusionary criteria as a positive step towards a common language between professionals and academics. However, some commentators recommend that criteria previously used to exclude a diagnosis of SLI should still be noted, but should no longer exclude diagnosis (Rutter 2014, Strudwick and Bauer 2014).

The evidence against using non-verbal IQ is discussed in some detail. Both lead articles and the commentators agree that requiring a gap between non-verbal IQ and language abilities (‘cognitive referencing’) should be dropped, as its use is ‘largely discredited’ (Bishop 2014), ‘conceptually unsound’ (Reilly *et al*. 2014a) and ‘misinformed’ (Leonard 2014). However, there is disagreement about whether there should be some minimal level of non-verbal ability, and if so, what that level should be. Hansson *et al*. (2014) state that a cut-off of 70 is used in most Swedish research on SLI.

In terms of what label to give to children with unexplained language problems, the articles and commentaries are more mixed in their views. However, in their response article, Reilly *et al*. (2014b) rule out three potential labels: *Language delay* because it ‘implies eventual catch-up in skills, which is not typically what is seen’ and is ‘often used to deny services to children’ (see also Wright 2014); *primary language impairment* because it is difficult to judge which condition is primary in a child with more than one impairment (Conti-Ramsden 2014) and it could be confused with primary school-age (Clark and Carter 2014); and *language disorder* because it yields too many results unrelated to children's unexplained language problems when entered as a search term (Bishop 2014).

Half of the commentators are in favour of dropping the term SLI to reflect the relaxation of exclusionary criteria which all agreed is required. But others feel that changing the label risks breaking the link with past research (Gallagher 2014, Rice 2014, Taylor 2014) and prefer to keep the term, but revise the meaning of the term ‘specific’ to mean ‘idiopathic’ (i.e., ‘of unknown origin’; Bishop 2014). However, concern is expressed that keeping the term ‘specific’ would encourage people to continue to use inappropriate exclusionary criteria.

Of the remaining possible terms, *language learning impairment* was viewed favourably by most, except parents (Hüneke and Lascelles 2014). The fewest objections were raised to *developmental language disorder*, where *disorder* is used to refer to conditions without obvious aetiology (Baird 2014), not to whether the child's language profile is ‘spikey’ or ‘flat’; a distinction which Reilly *et al*. (2014b) say ‘has no validity as an indicator of either aetiology or prognosis’.

Ultimately, Reilly *et al*. (2014b) argue in their response article that we need a diagnostic label which works for services, families and individuals. This is an ambitious goal, but in order to achieve it, the authors (and several of the commentators) call for an international and multidisciplinary panel to be formed that should aim to build consensus about first the diagnostic criteria and second the diagnostic label. This panel should take account of the views of families and people with language problems and policy-makers and could produce a position statement on the issue. In the meantime, we would like to solicit the views of readers of this special issue and encourage you to join in the discussion via http://www.rcslt.org/news/news/2014_news_archive/ijlcd_discussion_forum.

It has been a great pleasure to work with Dorothy Bishop, Sheena Reilly and Bruce Tomblin while editing this special issue and also to work with the 32 commentators who between them provided 20 wonderfully insightful commentaries. I thank them all for meeting extremely tight deadlines to enable this project to come to fruition under a year from its inception.
